# Thin PDS Foils Represent an Equally Favorable Restorative Material for Orbital Floor Fractures Compared to Titanium Meshes

**DOI:** 10.3390/tomography9040121

**Published:** 2023-08-16

**Authors:** Juergen Taxis, Lena Ungerboeck, Constantin Motel, Alexander W. Eckert, Natascha Platz Batista da Silva, Felix Nieberle, Nils Ludwig, Johannes K. Meier, Tobias Ettl, Torsten E. Reichert, Steffen Spoerl

**Affiliations:** 1 Department of Cranio- and Maxillofacial Surgery, University Hospital Regensburg, Franz-Josef-Strauß-Allee 11, 93053 Regensburg, Germany; lena.ungerboeck@stud.uni-regensburg.de (L.U.); felix.nieberle@stud.uni-regensburg.de (F.N.); nils.ludwig@ukr.de (N.L.); johannes.meier@ukr.de (J.K.M.); tobias.ettl@ukr.de (T.E.); torsten.reichert@ukr.de (T.E.R.); steffen.spoerl@ukr.de (S.S.); 2 Department of Cranio- and Maxillofacial Surgery, Paracelsus Medical University Nuremberg, Breslauer Straße 201, 90471 Nuremberg, Germany; c.motel@hotmail.de (C.M.); alexander.eckert@klinikum-nuernberg.de (A.W.E.); 3 Department of Radiology, University Hospital Regensburg, Franz-Josef-Strauß-Allee 11, 93053 Regensburg, Germany; natascha.platz-batista-da-silva@ukr.de

**Keywords:** orbital floor fracture, orbital volume analysis, three-dimensional measurement, PDS foil, titanium mesh

## Abstract

Orbital floor fractures (OFFs) are common injuries of the midface and may result in long-term complications. The aim of this study was to compare two restoration materials, PDS foils and titanium meshes, with regards to (1) clinical outcome and (2) reduction in orbital volume. The monocentric discovery cohort was analyzed retrospectively and included 476 patients with OFFs treated between 2010 and 2020. A subcohort of 104 patients (study cohort) with isolated OFFs and available high-resolution imaging material was used for volume measurements. Postoperative complications were not significantly different between patients treated with different restoration materials. Prevalence of revision surgery was significantly higher in patients treated with thick PDS foils (25 mm). OFFs treated with PDS foils and titanium meshes showed a significant reduction in orbital volume (*p* = 0.0422 and *p* = 0.0056, respectively), however, this volume decrease was significantly less pronounced in patients treated with PDS foils alone (*p* = 0.0134). Restoration using PDS foil in an isolated OFF reduces the orbital volume to a lesser extent than titanium mesh. Class III patients according to the classification of Jaquiéry with a missing bony ledge medial to the infraorbital fissure particularly benefit from restoration with PDS foils due to a lower reduction in the orbital volume. Regarding short- and long-term postoperative complications, a PDS foil thickness of 0.15 mm appears equivalent to titanium mesh in the treatment of OFFs.

## 1. Introduction

For a long time, no consensus had been found about fundamental factors for surgeons to determine preoperatively which patient suffering from isolated orbital floor fracture (OFF) would benefit from surgery [1,2,3]. OFF is a common injury of the face, accounting for about 15% of all facial skeleton fractures, and can lead to enophthalmos, limited ocular movement as a sign of muscle entrapment, diplopia, pain with directional gaze, and oculocardiac reflex [4,5]. While there has not yet been a definite unification of therapy guidelines, in cases where there is early enophthalmos greater than 2 mm, defects of the orbital floor larger than 2 cm^2^, or computed tomographic (CT) evidence of entrapment accompanied by symptomatic diplopia, gaze restriction, or non-resolving oculocardiac reflex, early surgical intervention is highly recommended [2,3,6]. In particular, enophthalmos, an indicator of enlarged orbital volume, is a critical and deceptive factor to consider regarding therapeutic options, as hemorrhage and edema of orbital tissue might initially conceal enlarged volume or even lead to proptosis [2,7,8]. This highlights the importance of accurate and early detection of orbital volume changes as a critical factor in determining the type of intervention [2,9].

Historically, the only means of adequately evaluating orbital volumes has been by measuring enophthalmos, as no other diagnostic tools have been available. Further complicating the decision-making, no uniform consensus exists about the material to use in reconstruction if surgery is required [10]. There is a wide range of materials in clinical use, including titanium meshes, autologous bone grafts, autologous rib cartilage grafts, resorbable and non-resorbable alloplastic materials, and preformed or patient-specific implants (PSI). No official guidelines have been formulated so far [11,12]. Mok et al., have outlined the ideal characteristics that materials used for reconstruction should possess. It is imperative that these materials are resorbable, osteoconductive, resistant to infection, minimally reactive, preventative of capsule formation, affordable, easily accessible, with a half-life that allows for significant bony ingrowth [12]. As no material meets all these requirements simultaneously, surgeons still base their choice on personal preference, experience, and training [13].

Ellis et al., and Tan et al., showed that orbits reconstructed with titanium meshes resulted in better overall reconstruction than those with bone grafts [14]. Further, Ellis et al., and Messo et al., evaluated titanium meshes as superior to other alloplastic materials like polydioxanone (PDS), silicone, or Teflon. They reported sterile inflammation in reconstruction with PDS, leading to granulomas and soft tissue scarring and failing to maintain the normal contour of the orbit, which was not apparent in titanium mesh reconstruction [15]. Contrary to this observation, Seifert et al., mentioned inflammatory reactions caused by titanium meshes and an overall association with significantly more postoperative complications, such as infections, extrusion, and residual diplopia, favoring PDS foil reconstruction [16]. Similar results were seen by Holtmann et al., who compared the reconstruction of OFFs with 0.15 mm PDS foil, 0.25 mm PDS foil, and titanium mesh and found 0.15 mm PDS foil yielded the best results in orbital floor defects [10].

Even though Holtmann et al., and Seifert et al., included sufficient numbers of study participants, 507 and 1594, respectively, only defect sizes were calculated, and no volume measurements were taken to evaluate the volumetric dimensions of the affected orbits pre- and postoperatively [10,16]. To the best of our knowledge, only one recently published study by Radović et al., integrated this aspect of imaging in their evaluation of orbital floor defect treatment, despite including only 58 patients [17].

In summary, there are no official guidelines for isolated OFF treatment regarding clear cut-off values for trauma-induced orbital volume changes or a recommended material for orbital floor reconstruction. Therefore, the aim of this study was to compare orbital volumes of patients with isolated OFFs pre- and postoperatively, when reconstructed with either PDS foil or with titanium mesh. Furthermore, surgery-related parameters were evaluated regarding the supply material and thus the clinical outcome was assessed.

## 2. Materials and Methods

### 2.1. Patient Selection and Data Collection

**Figure 1:**
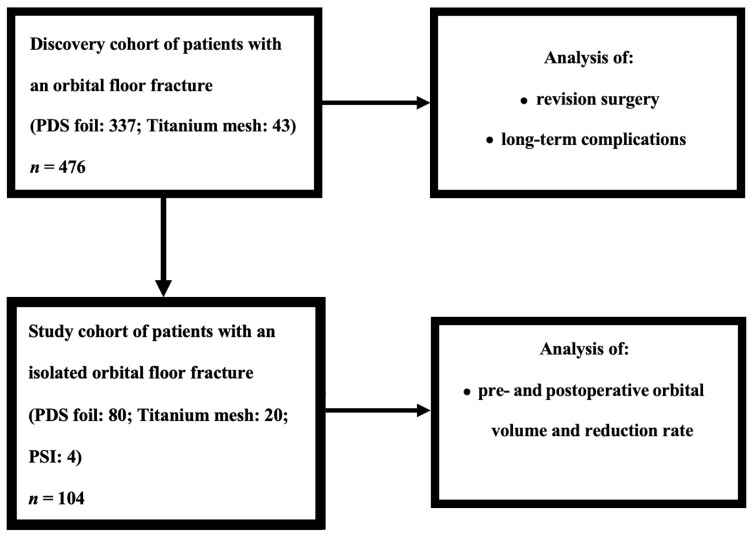
Flowchart of cohort breakdown into discovery and study cohorts.

This retrospective monocentric study was performed at the Department of Cranio- and Maxillofacial Surgery, University Hospital Regensburg, Germany. Initially, a total of 476 patients who underwent surgery for an OFF between the years 2010 and 2020 were included in the study (discovery cohort). As previously described in our multicenter study [18], clinical data such as gender and age, cause of fracture, timing and duration of surgical intervention, type of restoration, and length of hospital stay were collected. When a PDS foil was used as the supply material, the foil thickness was included in the analysis. Preoperatively as well as in the postoperative course, patients were examined and evaluated by ophthalmologists for the presence of diplopia, motility restrictions of the bulb, and exophthalmos. As mentioned before, certain requirements needed to be met to assess the size of the orbital volumes [18]. These included the presence of an isolated OFF and access to at least two high-quality CT scans, one before the operation and one afterward. The scans and measurements used for data collection included coronal, axial, and sagittal midface reformations (bone windows) with a 0.75 mm slice thickness. Based on the availability of the data, a subcohort from the discovery cohort was created, resulting in a study cohort that included 104 patients and was used for the calculation of the orbital volumes (Figure 1). In these patients, OFF classification was further performed according to Jaquiéry et al. [19].

### 2.2. 3D Model Preparation

**Figure 2:**
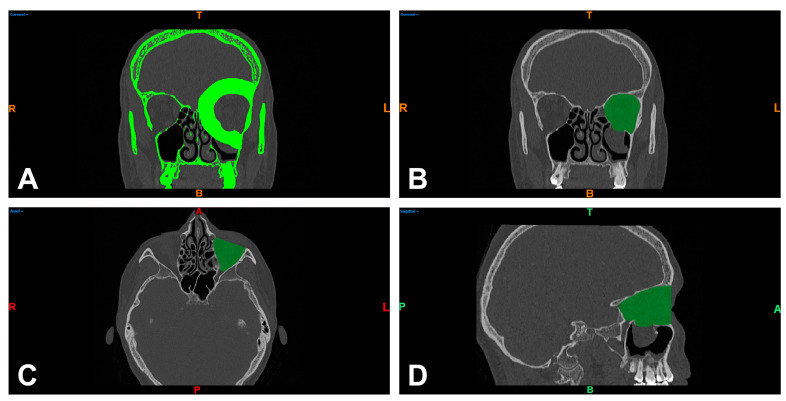
Separation of bony structures via thresholding of Hounsfield units and virtual tissue dissection of the left orbit in the coronal reformation of the midface (bone window) (**A**) and visualization of the enclosed orbital volume in the coronal (**B**), axial (**C**), and sagittal slice images (**D**). R = right; T = top; L = left; colored B = bottom; colored A = anterior; P = posterior.

From patients with an isolated OFF, DICOM data from CT scans were imported from the study cohort into Mimics imaging software (Mimics Innovation Suite 21.0; Materialise, Leuven, Belgium) for further processing. In accordance with our preliminary work on the 3D measurement of OFFs, the CT images were color coded to separate the affected areas in the trauma zone as well as the orbit from the unaffected parts of the scanned midface [18]. The thresholding of Hounsfield units (HU) was performed for differentiation, as well as virtual tissue dissection using the mentioned software, so that the bony orbit was separated from the remaining skeletal elements. A separate working mask was used to manually close the bony orbital volume directly below the orbital floor and the defect (Figure 2A). In conclusion, a 3D model of the bony orbit was built and exported as an STL (Standard Triangle Language) file for 3D analysis.

### 2.3. Orbital Volume Measurement

**Figure 3:**
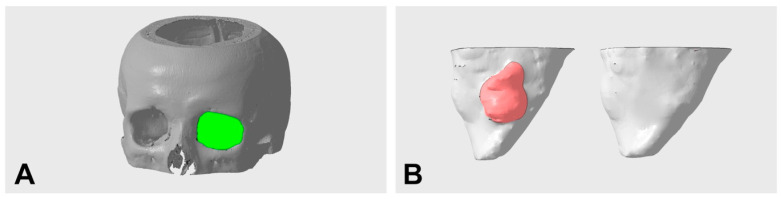
Representative illustration of the orbital volume defined for measurement (**A**) and segmented pre- and postoperative orbital volumes with defect corrected using PDS foil (**B**) (left preoperative with defect region in red; right postoperative without defect).

To obtain pre- and postoperative orbital volume measurements, the exported STL files were imported into Netfabb software (Netfabb Premium 2020; Autodesk Inc., San Rafael, CA, USA). Using a Boolean operation, the model of the 3D orbit was subtracted from a separate solid auxiliary model. The resulting new model showed, in the form of the difference between both models, the orbital volume and the remnants of the auxiliary model lying beyond the bony orbit. These remnants, which were not within the searched volume, were manually removed. The ventral boundary of the orbital volume was defined as the margo infra- and supraorbitalis. The dorsal boundary was the canalis opticus foramen. Figure 2B–D and Figure 3A show representative images of the orbital volume thus included. The preparation and assessment of the orbital volumes described in this way were performed independently by two investigators (J.T. and L.U.).

### 2.4. Statistical Analysis

Statistical data analysis was performed using IBM SPSS Statistics 26.0 (IBM Corp., Armonk, NY, USA) and GraphPad Prism 9.0 (GraphPad Software, La Jolla, CA, USA). The median (MED), mean (MV), and standard deviation (SD) of the measured preoperative and postoperative orbital volumes were calculated. Univariate analysis was performed using a chi-square test to compare distinct reconstructive materials used with surgery-related variables. Mann–Whitney tests were used for comparisons depending on the supply type of OFFs, and Wilcoxon matched-pairs signed rank tests were used to analyze the differences between the calculated pre- and postoperative orbital volumes. *p*-values < 0.05 were considered significant.

## 3. Results

### 3.1. Characterization of the Patient Cohorts

Surgically treated over a 10-year period, 476 patients with orbital floor fracture were included in the discovery cohort. Of these, 138 patients were female (29%) and 338 male (71%), with an overall mean age of 45.52 years (range: 6–92 years). A fall event (170 cases; 35.9% of the cohort) was the most common cause of fracture. Furthermore, 110 cases caused by a rough offense (23.3%) and 87 cases caused by a sports accident (18.4%) were also included. A total of 49 cases had a traffic accident as the cause of fracture (10.4%), followed by 11 cases caused by a horse kick (2.3%) and 46 cases (9.7%) with another cause of fracture. On average, surgical treatment was performed after 12.57 days (range: 0–35 days) and surgery lasted 78.02 min (range: 15–550 min). The most common treatment was PDS foil (337 cases; 70.8%), followed by titanium mesh (43 cases; 9%), PSI (5 cases; 1.1%), and maxillary sinus balloon (3 cases; 0.6%). A monocortical iliac crest bone was used in 2 patients (0.4%), only the orbital floor was reduced in 25 patients (5.3%), and no treatment or patients declined surgical treatment occurred in 61 cases (12.8%). In the case of treatment with a PDS foil, a foil thickness of 0.15 mm (57.57%) was used in 194 cases, and a thickness of 0.25 mm (42.43%) was used in 143 cases. The average inpatient stay of all surgically treated patients was 8.29 days (range: 1–61 days). Patient characteristics for the discovery cohort are shown in Table S1.

The study cohort included 104 patients who fulfilled the previously defined criteria. According to the classification of Jaquiéry et al. [19], 1 OFF was defined as class I (0.9%), 68 as class II (65.4%), and 35 as class III (33.7%). In total, 32 patients of the study cohort were female (30.8%) and 72 were male (69.2%), with an overall mean age of 47.79 years (range: 14–86 years). In the study cohort, the most common cause of fracture was also a fall event with 31 cases (29.8%), followed by rough offenses and traffic accident with 20 cases each (19.2% each). A further 11 patients (10.6%) had a sports accident, 3 patients (2.9%) suffered a horse kick, and 19 patients (18.3%) had another cause for the fracture. In this group, surgical treatment occurred after an average of 4.47 days (range: 0–22 days) and lasted a mean of 104.91 min (range: 23–376 min). In the study cohort, 80 patients were treated with a PDS foil (76.9%), 20 patients with a titanium mesh (19.2%), and 4 patients with a PSI (3.8%). The PDS foil thickness used was 0.25 mm in 41 cases (51.2%) and 0.15 mm in 39 cases (48.8%). The total inpatient stay of surgically treated patients averaged 11.13 days (range: 3–61 days), and the surgical procedure was often delayed by a few days due to posttraumatic swelling. Thus, the study cohort, as a subcohort of the discovery cohort, had a similar distribution of clinicopathological data and can be considered representative despite including a smaller number of patients. Clinicopathological data of the study cohort are presented in Table 1.

### 3.2. Clinical Outcome Parameters in Relation to Distinct Reconstructive Materials within the Discovery Cohort

The overall outcome of the surgical interventions with special regards to distinct reconstructive materials was analyzed within the discovery cohort. In the subgroups of patients treated with different types of PDS foil as well as titanium meshes (*n* = 380), we investigated patients’ individual outcomes with regards to the chosen material of orbital floor reconstruction. The need for a revision surgery did not differ between patients treated with a PDS foil and those having received a titanium mesh (*p* = 0.416; Figure S1A). Additionally, when focusing on long-term complications such as persistent diplopia, impairment of bulbar motility, and en- or exophthalmos after orbital floor reconstruction, a similar result was observed for both groups of reconstructive materials (*p* = 0.722; Figure S2).

However, when cases treated with a PDS foil were analyzed in detail, the prevalence of revision surgery was significantly higher when thicker PDS foils were chosen (0.15 vs. 0.25 mm; *p* = 0.005; Figure S1B).

### 3.3. Orbital Volume Analysis in the Study Cohort

**Figure 4:**
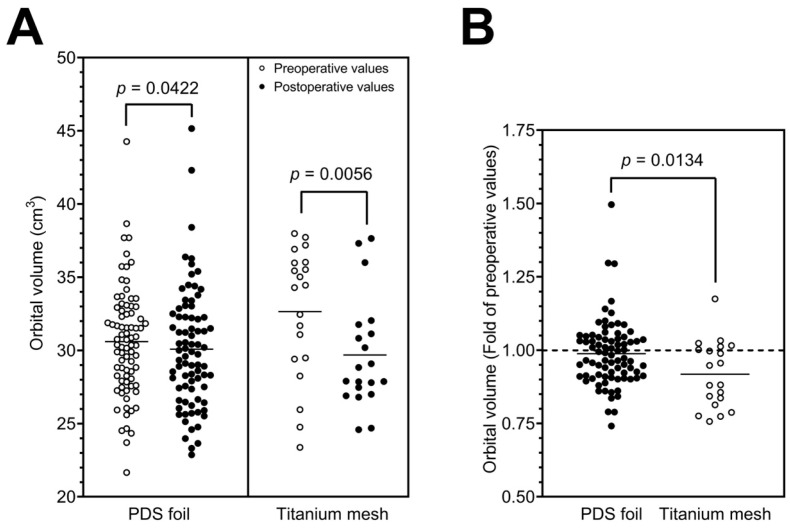
Pre- and postoperatively calculated orbital volumes with a statistically significant volume reduction for PDS foil and a highly significant reduction for titanium mesh (**A**). Volume reduction calculated for each patient using the fold change in preoperative values with a statistically significant difference between both restorations (**B**).

**Figure 5:**
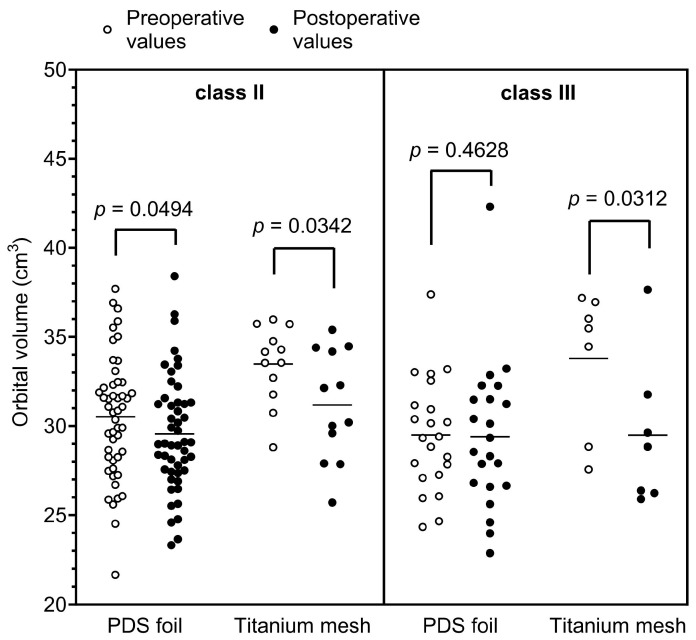
Pre- and postoperatively calculated orbital volumes according to the classification of Jaquiéry et al., in class II and III patients [19].

To evaluate the differences between the groups further, orbital volumes were calculated pre- and postoperatively in a subcohort of patients (study cohort; *n* = 104). When fractures were treated with PDS foil, the calculated preoperative orbital volumes had an average value of 30.60 cm^3^ with a standard deviation of 3.75 cm^3^ (minimum 21.66 cm^3^ and maximum 44.27 cm^3^), and the postoperative orbital volumes had an average value of 30.09 cm^3^ with a standard deviation of 4.07 cm^3^ (minimum 22.87 cm^3^ and maximum 45.16 cm^3^). In contrast, orbital floors restored with titanium mesh yielded an average preoperative volume of 32.65 cm^3^ with a standard deviation of 4.47 cm^3^ (minimum 23.38 cm^3^ and maximum 37.99 cm^3^) and an average postoperative volume of 29.69 cm^3^ with a standard deviation of 3.76 cm^3^ (minimum 24.59 cm^3^ and maximum 37.65 cm^3^). Here, the paired *t*-test revealed a highly statistically significant difference between the pre- and postoperatively measured volumes for restorations with titanium meshes (*p* = 0.0056, Figure 4A; Table 2) and a statistically significant difference for restorations with PDS foils (*p* = 0.0422, Figure 4A; Table 2). Figure 3B shows a representative example of the segmented preoperative and postoperative orbital volume, volume reduction, and restoration of the defect using a PDS foil. To further investigate the aspect of volume reduction with the respective surgically placed material, the fold of the preoperative orbital volume was calculated for each individual patient. This calculation revealed a 0.98-fold difference between the post- and preoperative measurements for restorations with PDS foils (SD: 1.14-fold; minimum 1.06-fold and maximum 1.02-fold). Restorations with titanium meshes showed a 0.91-fold difference between post- and preoperative measurements (SD: 0.84-fold; minimum 1.05-fold and maximum 0.99-fold). The folds of preoperative orbital volumes were significantly different (*p* = 0.0134; Figure 4B). In summary, these data demonstrate that orbital volumes are more reduced when titanium meshes are used than when PDS foils are used. All results are presented in Table 2. To further validate these results, subgroups were divided according to the classification of Jaquiéry et al., and analyzed [19]. Most patients belonged to class II or class III; thus, all patients had a defect of the orbital floor and/or of the medial wall with fracture areas larger than 2 cm^2^. The difference between class II and III is that the bony ledge is preserved at the medial margin of the infraorbital fissure in class II patients, and the bony ledge is missing medial to the infraorbital fissure in class III patients. In class II patients, postoperative orbital volume values were significantly decreased for PDS foils and titanium meshes in a comparable manner (*p* = 0.0494 and *p* = 0.0342, respectively, Figure 5). In class III patients, there was no significant reduction in orbital volumes with regards to PDS foils; however, a significant reduction was observed with regards to titanium meshes (*p* = 0.0312, Figure 5). These results indicate that patients with a missing bony ledge medial to the infraorbital fissure benefit from reconstruction with PDS foils due to smaller reduction in orbital volumes.

Four patients of the study cohort received PSIs, and respective results are presented in Figure S3. Although differences were not statistically significant due to the small number of patients, there was a trend of postoperative volume reduction that was more pronounced compared to that seen with PDS foils. Patients exhibited a 0.83-fold difference between pre- and postoperative values (SD: 3.25-fold; minimum 0.74-fold and maximum 0.89-fold; Table S2).

## 4. Discussion

In the present study, we investigated the clinical outcome of surgically treated OFFs and specifically focused on different restoration materials. Furthermore, we evaluated the volume change in the affected orbit in a fraction of these patients in relation to treatment with PDS foil or titanium mesh. We were able to demonstrate that the frequency of revision surgery was not statistically significant when using PDS foil or titanium mesh for fracture treatment (*p* = 0.416). Analogous, long-term postoperative complications such as persistent diplopia, bulbar motility impairment, and en- or exophthalmos were equally rare when comparing patients treated with these types of materials (*p* = 0.722). When considering only the PDS foils and specifically the foil thickness, there was a statistically significant association with the occurrence of revision surgery and the use of a thicker foil of 0.25 mm (*p* = 0.005). This is in agreement with the results of Holtmann et al. [10], who also concluded that thin PDS foils of 0.15 mm in thickness provided the best clinical results.

Regarding the orbital volume change, we demonstrated that the volume in restorations with PDS foils was reduced from an average of 30.60 cm^3^ to 30.09 cm^3^, thus indicating a 0.98-fold decrease. In contrast, restorations with titanium mesh reduced the orbital volume from an average of 32.65 cm^3^ to 29.69 cm^3^, representing a 0.91-fold reduction in the fractured orbit volume. The reduction in the orbital volume by insertion of a titanium mesh was statistically highly significant (*p* = 0.0056) in contrast to the volume change with a PDS foil (*p* = 0.0422), which was at the border of statistical significance. After subgrouping the study cohort according to Jaquiéry et al., it was shown that patients with a class II OFF were equally associated with a significant reduction in the orbital volume after restoration with titanium meshes or PDS foils (*p* = 0.0494 and *p* = 0.0342, respectively). In class III patients, however, only reconstructions with titanium meshes significantly reduced the orbital volume (*p* = 0.0312), which could be due to the size of the defect and the thickness of the mesh material. On average, the pre- and postoperative volumes showed a similar size range to those reported in previous studies [20,21].

PDS foils are easier to customize relative to the defect, as they can be easily shaped and cut to fit the contour of the patient’s individual orbital defect [22,23]. In contrast, titanium meshes must be bent or cut during surgery and placement in the deep orbital conus proves to be difficult [22,23]. Accurate restoration of orbital volume represents a crucial aspect in the treatment of OFFs and avoidance of long-term complications [17,24]. However, in agreement with the results of Schönegg et al., we could not find a statistically significant correlation between short- or long-term complications and orbital volume change due to the different fitting materials [20]. This again is in agreement with the study of Baek et al., who observed that resorbable and non-resorbable implants were equivalent with regards to postoperative risks [25]. Therefore, it can be argued that PDS foils are equal to titanium meshes as a restorative material in most cases of isolated OFFs.

Nevertheless, our study has some limitations. Firstly, the unequal distribution of restorations with PDS foils and titanium meshes should be mentioned; this was due to the individual preference of the surgeons and primarily had no medical reasons. As a result, the surgeons were very well versed in the surgical use of PDS foils, which may have led to the lower incidence of long-term complications presented here and therefore may have caused bias in the results. The fact that particularly large OFFs could only be treated with a titanium mesh was not considered in this work. Furthermore, the volume measurements were evaluated independently by two examiners but were mostly determined manually, which may invite a certain susceptibility to error. In this regard, the use of machine learning and neural networks to establish fully automated orbital volume measurement in diagnosis and postoperative control represents an interesting approach for future studies.

## 5. Conclusions

In summary, our results show that PDS foils reduce the orbital volume to a lesser extent than titanium meshes in the restoration of isolated OFFs. In general, similar good clinical results in terms of long-term complications and revision surgery were achieved between the restoration of OFFs with PDS foils, especially with the foil thickness 0.15 mm, and that performed with titanium meshes. In this respect, PDS foils with a thickness of 0.15 mm are shown to be equal to titanium meshes for the treatment of OFFs and more advantageous in the treatment of isolated OFFs.

## Figures and Tables

**Table 1:** Clinicopathological characteristics of the study cohort.

Category	Study Cohort
	Total (*n* = 104)
Gender:	
Female	32 (30.8%)
Male	72 (69.2%)
Age (MV in years)	47.79 (14 to 86)
Cause of fracture:	
Rough offense	20 (19.2%)
Fall	31 (29.8%)
Sports accident	11 (10.6%)
Traffic accident	20 (19.2%)
Horse kick	3 (2.9%)
Other	19 (18.3%)
Surgery after fracture (MV in days)	4.47 (0 to 22)
Surgery duration (MV in minutes)	104.91 (23 to 376)
Classification according to Jaquiéry et al.:	
Class I	1 (0.9%)
Class II	68 (65.4%)
Class III	35 (33.7%)
Supply type:	
PDS foil	80 (76.9%)
Titanium mesh	20 (19.2%)
PSI	4 (3.8%)
Monocortical iliac crest	-
Maxillary sinus balloon	-
Only reduction	-
Untreated or refused supply	-
PDS foil thickness (mm):	
0.15	39 (48.8%)
0.25	41 (51.2%)
Inpatient stay (MV in days)	11.13 (3 to 61)

MV = mean value; PDS = polydioxanone; PSI = patient-specific implant. The range of age, surgery after fracture, surgery duration and inpatient stay is given in brackets.

**Table 2:** Pre- and postoperative orbital volumes in isolated OFFs and fold change in preoperative values.

*n* = 104	PDS Foil			Titanium Mesh		
	Volume (cm^3^)		Fold Change in Preoperative Values	Volume (cm^3^)		Fold Change in Preoperative Values
	Preoperative	Postoperative		Preoperative	Postoperative	
Mean	30.60	30.09	0.98	32.65	29.69	0.91
Median	30.57	29.83	0.98	34.37	28.37	0.83
SD	3.75	4.07	1.14	4.47	3.76	0.84
Minimum	21.66	22.87	1.06	23.38	24.59	1.05
Maximum	44.27	45.16	1.02	37.99	37.65	0.99
*p*-value	0.0422			0.0056		

PDS = polydioxanone; SD = standard deviation.

## Data Availability

Data can be obtained by scientists conducting work independently from the industry upon request. Data are not stored on publicly available servers.

## Supplementary Materials

The following supporting information can be downloaded at: https://www.mdpi.com/article/10.3390/tomography9040121/s1, Figure S1: Impact of the chosen supply material (A) as well as the PDS foil thickness (B) on the need of a revision surgery; Figure S2: Impact of the chosen supply material on long-term complications when restoration was performed using PDS foil or titanium mesh; Figure S3: Pre- and postoperatively calculated orbital volumes with statistically non-significant volume reduction with PSI (A) and volume reduction calculated for each patient using the fold change in preoperative values (B); Table S1: Clinicopathological characteristics of the discovery cohort; Table S2: Pre- and postoperative orbital volumes in isolated OFFs and fold change in preoperative values in PSI treatment.
