# Supplementary material for: Thin PDS Foils Represent an Equally Favorable Restorative Material for Orbital Floor Fractures Compared to Titanium Meshes
Source: Tomography. 2023 Aug 16;9(4):1515–25. doi: 10.3390/tomography9040121 (PMC10458727; doi:10.3390/tomography9040121)
Supplement: Supplementary file 1 [file tomography-09-00121-s001.zip › Tables_S1.pdf]

Table S1. Clinicopathological characteristics of the discovery cohort.

Category	Discovery cohort
	**Total** (*n* = 476)
Gender:	
Female	138 (29 %)
Male	338 (71 %)
Age (MV in years)	45.52 (6 to 92)
Cause of fracture:	
Rough offence	110 (23.3 %)
Fall	170 (35.9 %)
Sports accident	87 (18.4 %)
Traffic accident	49 (10.4 %)
Horse kick	11 (2.3 %)
Other	46 (9.7 %)
Surgery after fracture (MV in days)	12.57 (0 to 35)
Surgery duration (MV in minutes)	78.02 (15 to 550)
Supply type:	
PDS foil	337 (70.8 %)
Titanium mesh	43 (9 %)
PSI	5 (1.1 %)
Monocortical iliac crest	2 (0.4 %)
Maxillary sinus balloon	3 (0.6 %)
Only Reduction	25 (5.3 %)
Untreated or refusal of supply	61 (12.8 %)
PDS foil thickness: (mm)	
0.15	194 (57.57 %)
0.25	143 (42.43 %)
Inpatient stay (MV in days)	8.29 (1 to 61)

MV = mean value; PDS = polydioxanone; PSI = patient specific implant. The range of age, surgery after fracture, surgery duration and inpatient stay is given in brackets.

Table S2. Pre- and postoperative orbital volumes in isolated OFFs and fold of preoperative values in PSI treatment.

n = 4	**PSI**		
	**Volume (cm^3)**		**Fold of preoperative values**
	**preoperative**	**postoperative**	
Mean	36.43	30.12	0.83
Median	36.42	30.44	0.84
SD	0.89	2.89	3.25
Minimum	35.48	26.38	0.74
Maximum	37.39	33.22	0.89
p-value	0.1250		

PSI = patient specific implant; SD = standard deviation
