# Supplementary figures and images for: Thin PDS Foils Represent an Equally Favorable Restorative Material for Orbital Floor Fractures Compared to Titanium Meshes
Source: Tomography. 2023 Aug 16;9(4):1515–25. doi: 10.3390/tomography9040121 (PMC10458727; doi:10.3390/tomography9040121)

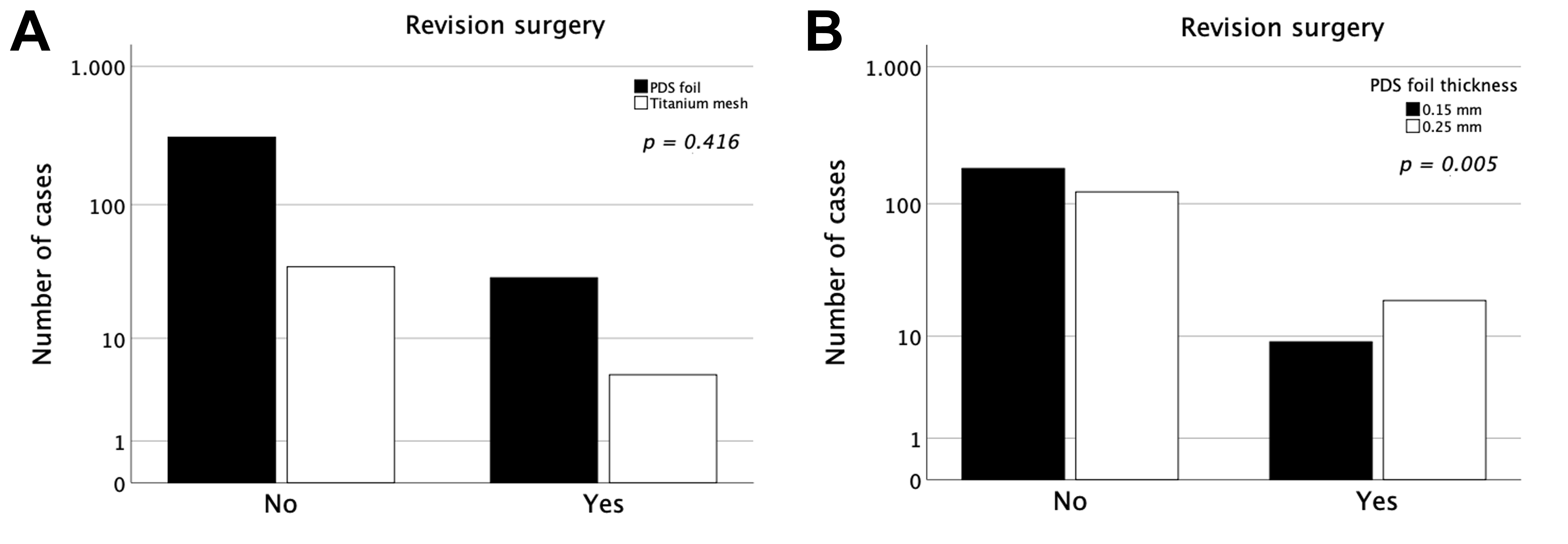

Supplement: Supplementary file 1 [file tomography-09-00121-s001.zip › Figure_S1.jpg]

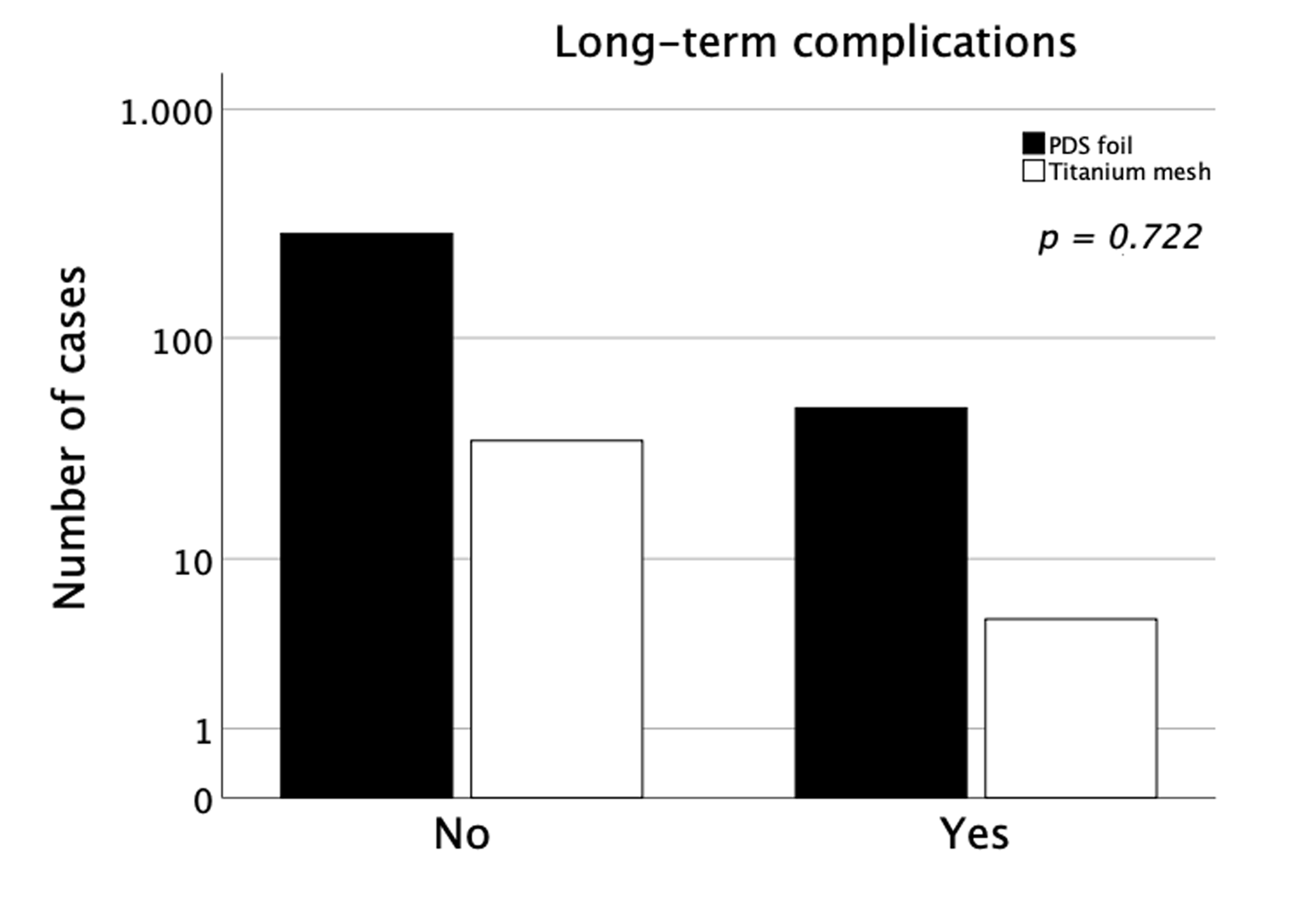

Supplement: Supplementary file 1 [file tomography-09-00121-s001.zip › Figure_S2.jpg]

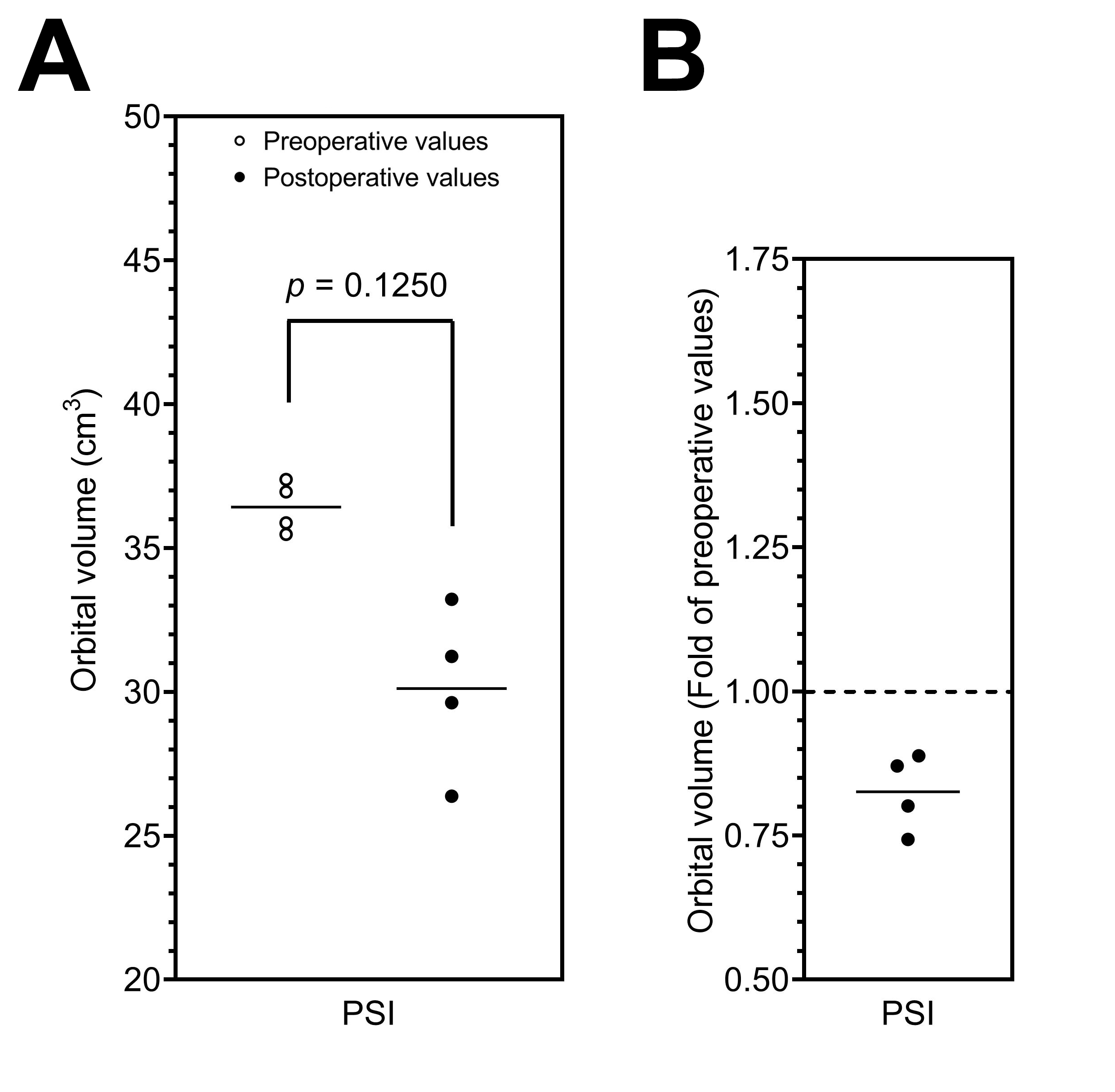

Supplement: Supplementary file 1 [file tomography-09-00121-s001.zip › Figure_S3.jpg]
